# Neutropenic Enterocolitis and Sepsis: Towards the Definition of a Pathologic Profile

**DOI:** 10.3390/medicina57060638

**Published:** 2021-06-20

**Authors:** Giuseppe Bertozzi, Aniello Maiese, Giovanna Passaro, Alberto Tosoni, Antonio Mirijello, Stefania De Simone, Benedetta Baldari, Luigi Cipolloni, Raffaele La Russa

**Affiliations:** 1 Section of Legal Medicine, Department of Clinical and Experimental Medicine, University of Foggia, Ospedale Colonnello D’Avanzo, Viale Europa 12, 71100 Foggia, Italy; giuseppe.bertozzi@unifg.it (G.B.); stefania.desimone@unifg.it (S.D.S.); luigi.cipolloni@unifg.it (L.C.); 2 Department of Surgical Pathology, Medical, Molecular and Critical Area, Institute of Legal Medicine, University of Pisa, 56126 Pisa, Italy; aniellomaiese@msn.com; 3 Fondazione Policlinico Universitario “A. Gemelli” IRCCS, 00168 Rome, Italy; passaro.giovanna@gmail.com; 4 CEMAD Digestive Disease Center, Fondazione Policlinico Universitario “A. Gemelli” IRCCS, Università Cattolica del Sacro Cuore, 00168 Rome, Italy; alberto.tosoni@policlinicogemelli.it; 5 Department of Medical Sciences, IRCCS Casa Sollievo della Sofferenza, 71013 San Giovanni Rotondo, Italy; a.mirijello@operapadrepio.it; 6 Department of Anatomical, Histological, Forensic and Orthopedic Sciences, Sapienza University of Rome, 00186 Rome, Italy; benedetta.baldari@uniroma1.it

**Keywords:** neutropenic enterocolitis, sepsis, chemotherapy-induced damage

## Abstract

*Background:* Neutropenic enterocolitis (NE), which in the past was also known as typhlitis or ileocecal syndrome for the segment of the gastrointestinal tract most affected, is a nosological entity that is difficult to diagnose and whose pathogenesis is not fully known to date. Initially described in pediatric patients with leukemic diseases, it has been gradually reported in adults with hematological malignancies and non-hematological conditions, such as leukemia, lymphoma, multiple myeloma, aplastic anemia, and also myelodysplastic syndromes, as well as being associated with other immunosuppressive causes such as AIDS treatment, therapy for solid tumors, and organ transplantation. Therefore, it is associated with high mortality due to the rapid evolution in worse clinical pictures: rapid progression to ischemia, necrosis, hemorrhage, perforation, multisystem organ failure, and sepsis. *Case report*: A case report is included to exemplify the clinical profile of patients with NE who develop sepsis. *Literature Review*: To identify a specific profile of subjects affected by neutropenic enterocolitis and the entity of the clinical condition most frequently associated with septic evolution, a systematic review of the literature was conducted. The inclusion criteria were as follows: English language, full-text availability, human subjects, and adult subjects. Finally, the papers were selected after the evaluation of the title and abstract to evaluate their congruity with the subject of this manuscript. Following these procedures, 19 eligible empirical studies were included in the present review. *Conclusions*: Despite the recent interest and the growing number of publications targeting sepsis and intending to identify biomarkers useful for its diagnosis, prognosis, and for the understanding of its pathogenesis, and especially for multi-organ dysfunction, and despite the extensive research period of the literature review, the number of publications on the topic “neutropenic enterocolitis and sepsis” appears to be very small. In any case, the extrapolated data allowed us to conclude that the integration of medical history, clinical and laboratory data, radiological imaging, and macroscopic and histological investigations can allow us to identify a specific pathological profile.

## 1. Introduction

Neutropenic enterocolitis (NE), as the phrase used to identify it suggests, is a severe inflammatory bowel disease that occurs in neutropenic patients. It is also known as ileocecal syndrome or “typhlitis”, from the Greek word “typhon”, used to indicate caecum or cecitis, since this is the site of the organism most frequently affected; it was a clinical entity initially described in pediatric leukemia patients. However, over the years, the diagnoses of adult subjects with neoplasms have increased (mainly hematological diagnoses, such as leukemia, lymphoma, multiple myeloma, aplastic anemia, and even myelodysplastic syndromes), as well as other immunosuppressive causes such as AIDS, therapy for solid tumors, and organ transplantation. However, since different tracts of the gastrointestinal (GI) system could be involved, it was considered more appropriate to use the definition of neutropenic enterocolitis [1,2,3].

The incidence of NE varies between studies. In a systemic review conducted by Gorschlüter et al., the incidence rate of 21 studies was 5.3% in patients hospitalized for hematologic malignancies, high-dose chemotherapy for solid tumors, or aplastic anemia, while another cohort study found it in 3.5% of 317 severely neutropenic patients [4,5].

The incidence of NE has increased with the increasing use of intensive chemotherapy [1]. Gastrointestinal toxicity, in fact, is a common complication of cytotoxic cancer chemotherapy. Currently available cytotoxic drugs do not discriminate between cancer cells and rapidly dividing normal cells. The toxicity of anticancer treatment will continue to be a significant problem until highly selective therapies for malignant cells are developed [6]. Combination regimens are often the standard treatment. The rapid extension of available antineoplastic drugs, however, has also underscored the urgent need for clinicians to better understand and detect the acute and late toxicity spectrum of these regimens.

In fact, exposure to cytotoxic drugs has been called into question as to the main mechanism in the pathogenesis of NE, although currently it is not yet fully understood. One of the mechanisms is the onset of mucositis with consequent interruption of the mucous barrier, which allows for bacterial translocation from the intestine. This mechanism is supported by histological findings of intestinal wall edema, swollen blood vessels, and mucosal surface rupture [7] with areas of ulceration and bleeding. Neutropenia further aggravates the risks, causing decreased immunity with the inability to control the transmural translocation of pathogens. There are also concerns that direct invasion of the interstitial wall by malignant cells may contribute to the disease. The cecum is more commonly involved in NE due to its distensibility and limited blood supply (elements which, by self-feeding, can in turn cause the clinical condition to worsen). 

Therefore, it is associated with high mortality due to the rapid evolution in worse clinical pictures: rapid progression to ischemia, necrosis, hemorrhage, perforation, and multisystem organ failure and sepsis, which is defined as infection-induced organ dysfunction or hypoperfusion abnormalities that predispose to septic shock and increased mortality in neutropenic settings [8]. It is underrecognized clinically, with the diagnosis often being made on post-mortem examination.

This review of the literature, focusing on the relationship between NE and sepsis in comparison with a clinical case, thus aims to favor the gnoseological diffusion of this nosological entity, to support the etiopathological mechanism, as proposed above, and to define its main characteristics for its diagnostic framework.

## 2. Case Report

**Figure 1:**
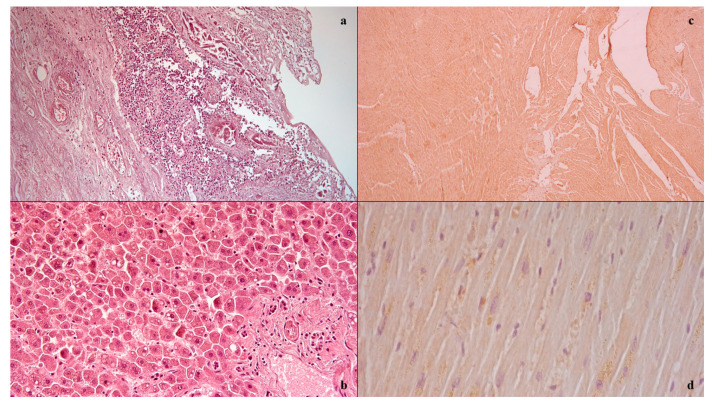
Histological investigation: mucosal and submucosal edema, and well-defined agglomerations of inflammatory cells in the context of the bowel wall (**a**); Councilman bodies of the liver (**b**); positive anti-IL15 (**c**) anti-TNF-alpha (**d**) antibody reactions on heart samples.

The case study involved a 56-year-old woman diagnosed with locally advanced infiltrating ductal carcinoma of the breast treated with chemotherapy according to the TAC scheme (Docetaxel, Doxorubicin, Cyclophosphamide). The blood tests at the beginning of chemotherapy were documented as follows: white blood cells 9630/mm^3^, of which 4860/mm^3^ were neutrophils. After 10 days from the start of chemotherapy, the woman in the case in question entered the local emergency department for abdominal pain refractory to medical pain-relieving therapy. Physical examination by the doctors documented the treatable abdomen on all quadrants with tenesmus, vomiting, and diarrhea, with the vital parameters of blood pressure at 110/70, 99% oxygen saturation, and body temperature at 38.0 °C. On laboratory tests, she had 560/mm^3^ of white blood cells, of which 290/mm^3^ were neutrophils. CT imaging showed the absence of pneumoperitoneum with a collapsed and thickened rectum, as well as fat stranding and intramural areas of low attenuation. A gastroenterological specialistic examination was also performed, which documented a smooth mucosa on rectal exploration, but with underlying layers there was increased consistency, circumferentially, and the presence of mucus bloody material into the lumen. During the diagnostic process, however, the patient’s condition suddenly worsened, due to the onset of hyperlactacidemic metabolic acidosis, respiratory insufficiency, and a tendency to hypotension despite the massive volume filling and the aggressive life support, and she died. The autopsy examination allowed us to detect, upon isolation of the intestinal tract between the ileocecal valve and the anus, the presence of a focal pattern of circumferential thickening and edema of the rectum-sigma mucosa, in the context of which it was possible to observe the presence of small yellow membranes. Upon cutting of the bowel wall, diffuse submucosal hemorrhages were also noted. The histological investigations (Figure 1) conducted on the organ samples allowed us to confirm the presence of mucosal and submucosal edema, and well-defined agglomerations of inflammatory cells in the context of the bowel wall as well as Councilman bodies were observed in the study of the liver in association with biliary stasis. The histological study was completed with immunohistochemical staining with positive anti-TNF-alpha and anti-IL15 antibody reactions on the heart samples [9,10]. The cause of death was attributed to sepsis and multi-organ failure.

## 3. Side Effects of Chemotherapy

Chemotherapy drugs can cause damage to the wall of the various tracts of the gastrointestinal (GI) system, according to multiple mechanisms. On the one hand, in fact, the damage can be mediated by a direct effect on the mucosa that can lead to inflammation, edema, ulceration, and atrophy of the same. On the other hand, these alterations cause, consequently, increased permeability of the mucosa which, in association with the immunosuppressive effect of the drugs themselves, predispose to an increased risk of transparietal infections, resulting in septicemia and shock, with consequent mucosal ischemia. This would trigger a self-sufficient vicious circle [11,12].

Among the drugs most implicated in mucosal damage, taxanes, which act by stabilizing microtubules and inhibiting cellular mitosis [13], have been linked to a broad spectrum of colitis. Specifically, it was difficult to compare the effects induced by docetaxel and paclitaxel in terms of toxicity, although docetaxel appears to be associated with more side effects than paclitaxel. The most frequent type of illness induced is ischemic colitis, clinically characterized by acute abdominal pain and associated neutropenia, fever, and/or diarrhea, with or without blood. This condition can develop into serious complications such as intestinal necrosis, colonic perforation, or typhlitis. Septicemia occurs frequently and the most common etiopathology is aerobic Gram-negative bacteria infection. The mucosal histopathological analysis is compatible with a significant component of inflammatory changes such as mucosal and submucosal edema, hemorrhage, acute inflammatory infiltrates, and mucosal ulcerations [14].

Platinum compounds follow, the best known of which is cisplatin. It works by binding inside cells to nucleophiles, such as DNA, RNA, and bases, to form adducts, which induce apoptosis [15]. Vomiting is the earliest GI symptom and is usually associated with a peak in the urinary metabolites of serotonin, suggesting a strong correlation between the release of serotonin with this agent and vomiting [16]. Oxaliplatin, on the other hand, has a large number of GI side effects: diarrhea and nausea, vomiting, stomatitis, dry mouth, melaena, bleeding, proctitis, and tenesmus.

Furthermore, doxorubicin is a drug belonging to the category of DNA intercalators, and it acts mainly by inhibiting DNA topoisomerase II and DNA replication through epigenetic mechanisms of DNA methylation [17]. Stomatitis has been reported in up to 80% of patients, in other cases, ulceration of the esophagus and colon has been described following its use.

On the other hand, 5-fluorouracil (5-FU) and methotrexate belong to the category of antimetabolites, i.e., analogs of folic acid, pyrimidine, or purines that induce cell death during the phase following incorporation into RNA and in DNA, thus inhibiting the synthesis of nucleic acid [18,19]. 5-FU, in detail, causes gastrointestinal side effects that can be serious and life threatening. Stomatitis and esophagopharyngitis are commonly observed during therapy, with ulceration and necrosis of the visceral wall. In fact, subsequent diarrhea can be bloody. Methotrexate, on the other hand, has a severe toxicity profile manifesting in myelosuppression, oropharyngeal ulceration, and diarrhea. Other frequently reported gastrointestinal side effects include stomatitis, hematemesis, melaena, and other types of bleeding. Extremely rare cases of toxic megacolon have been associated with the use of methotrexate.

## 4. Literature Review

Information Sources and Search: For this literature review the PubMed database was questioned on 30 April 2021. A primary selection was conducted with this search strategy: (neutropenic enterocolitis) showing 491 results. In order to focus on the link between neutropenic enterocolitis and sepsis, this search was narrowed to [(neutropenic enterocolitis) AND (sepsis)], resulting in 72 manuscripts.

Study Selection: The inclusion criteria were being in the English language and being published in a scholarly peer-reviewed journal. Full-length articles were preferred; duplicate manuscript or only abstract-available texts were excluded. Studies involving human targets were further selected. Moreover, the references of the selected articles were also reviewed.

**Figure 2:**
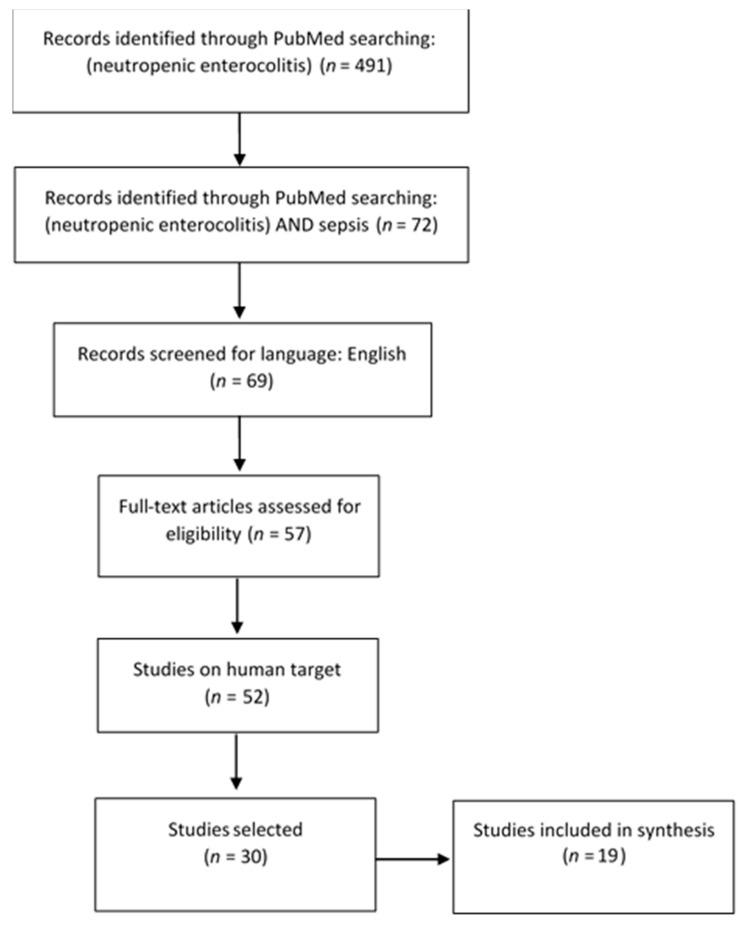
Review flow diagram, according to PRISMA guidelines.

Synthesis: Following these procedures, 30 eligible studies were included in present review. Then potentially relevant studies were further assessed, excluding other-than-neutropenic-enterocolitis entities causing sepsis [20,21,22,23,24,25,26], pediatric subjects [27], and clinical trials [28,29,30]. After this literature review process, 19 papers were selected (Figure 2).

## 5. Results

### 5.1. Etiology

From the literature review (Table 1), the most commonly isolated pathogens include *Pseudomonas aeruginosa*, *Escherichia coli*, *Bacillus cereus*, *Klebsiella* spp., *Enterococci*, *Clostridium* spp., and *Candida* spp. [31,32,33,34,35]. However, the organisms most frequently associated with sepsis or septic shock are Clostridium septicum, Citrobacter freundii, Stomatococcus mucilaginosus, and Stenotrophomonas maltophilia [36,37,38,39,40].

### 5.2. Kind of Neoplasia and Therapy

Regarding the history, all subjects identified from the reviewed literature were immunocompromised. In detail, the most frequently encountered pathologies were leukemia lymphoma [33,39,41,42,43]. Other pathological conditions associated with NE were found to be not only solid tumors, such as lung cancer and sarcoma [43], but also myelodysplasia or aplastic anemia [44].

Of those with a history of cancer and available clinical data, all had received chemotherapy in the previous month, particularly those agents that cause mucositis, such as taxanes [45].

Other documented chemotherapeutic agents included methotrexate, vincristine, cyclophosphamide, cytarabine, daunorubicin, cyclosporine, fludarabine, and/or doxorubicin or combinations, such as chemoradiotherapy (CRT) with 5-fluorouracil (5-FU) and mitomycin C [33,43,46].

### 5.3. Clinical and Imaging

Concerning the clinic complaints by the patient upon access to medical facilities, both general and specific GI involvement symptoms were reported for all confirmed NE patients when the next medical history was available. In particular, fever appeared in almost all patients, followed by pain or abdominal pain and diarrhea [47].

The reported laboratory tests identified as a common feature in all cases of major neutropenia with absolute neutrophil count <1500 mm^3^ [33,43]. Besides, subjects with absolute neutrophil count <500/mm^3^ more frequently had abdominal pain and higher fever as the main symptoms and more frequently encountered hemodynamic instability.

The diagnostic method most frequently used to reach the diagnosis was the CT scan of the abdomen, with signs of cecal inflammation and thickened edematous colon.

### 5.4. Macroscopic and Microscopic Features

Alterations were described in all segments of the GI including the ileus, left or right colon, or both, with involvement of different entities and considered mainly segmental or irregular (although diffuse or focal were also described) [33,34,35,36,37,38,39,40,41,42,43,44,45,46,47].

In order to macroscopically document these macroscopic alterations, endoscopic studies in NE cases have shown the following: mucosal ulceration, edema, erosions, pseudomembranes, nodularity, friability, or bleeding [33,43,48,49].

The histological features, on the other hand, of NE included the presence of intracellular organisms, necrosis, hemorrhage, ulceration, erosion, and pseudomembranes. However, a depleted inflammatory background was also described in some cases [33].

### 5.5. Treatment and Outcomes

In the study from Sachak et al. 79% of patients with histologically confirmed NE died after a median survival of 1 day [33].

Most of the symptomatic patients were treated with antibiotics or supportive perfusion therapy [42], and only a few benefited from granulocyte transfusion (GT) therapy or granulocyte colony-stimulating factor therapy [35]. This non-conservative approach demonstrated its efficacy in retrospective and prospective case series, but no studies have been successful in demonstrating its benefits from a statistically significant advantage in controlled clinical trials. It has also been associated with prophylactic bowel rest and total parenteral nutrition instituted at the beginning of further chemotherapy, with surgery delayed until complications appear [41]. 

NE-reported complications were as follows: sepsis, intra-abdominal abscesses, colon perforation, and pneumatosis intestinalis.

Patients with ongoing severe systemic sepsis and those with established complications (perforation, obstruction, hemorrhage, or abscess) require surgical intervention consisting of all necrotic material removal, usually performed with resective surgery of the affected segment [50,51]. According to Abu-Sbeih et al., all patients that required surgery had hematologic malignancies and absolute neutrophil counts <500/mm^3^ [43].

**Table 1:** Summary of the literature review.

Etiology	Kind of Neoplasia	Chemotherapy	Neutrophils	Imaging-CT	Macroscopic Examination	Microscopic Investigation
Echerichia coli [31,35,43,44,51,52]; Clostridium difficile [33,36,46,50,51]; Bacillus cereus [34]; Enterococcus sbb [33,35,43]; Clostridium septicum [33,36,37]; Stenotrophomonas maltophilia [38]; Citrobacter freundii [39]; Stomatococcos mucilaginosus [40]; Mucormycosis [42]; Fungi [33,34,35,50]; Viruses [33,35]; Aeromonas hydrophila [46]; Enterobacter cloacae [46]; Klebsiella pneumoniae [33,46]; Morganella morganii [52] Streptococcus oralis [52]	Leukemia/lymphoma [31,33,34,35,36,37,38,42,43,46,50,51,52], Lung carcinoma [33], Sarcoma [33], Breast cancer [39,45]; Vulvar cancer [47]	Cyclophosphamide [31,33,38,39,40,45,51]; Vincristine [31,33,40,51]; Methotrexate [33,40,51]; Cytarabine [33,36,42,51,52]; Daunorubicin [33]; Fludarabine [33]; Doxorubicin [33,45]; Idarubicina [34,42]; 6 thioguanine [36,40]; Daunorubicina [36,40,51]; Deoxycoformycin [37]; 5-fluorouracil [39,46]; Epirubicin [39]; Idarubicin [42]; Docetaxel [45]; Mitomycin C [47]	ANC = 1.2 × 10^9^/L [31]; ANC < 0.1 × 10^9^/L [35,44]; ANC < 0.5 × 10^9^/L [35,36]; ANC < 100 × 10 cells/mm^3^ [40,43]; 14.2% [42]; ANC = 500/mm^3^ [45]; ANC < 1000 cells/microL [50]	Thickened colon [33,34,42,44,45]; Small bowel dilatation [42];	Ulcerations [31,43]; Thickening and hemorrhagic walls [36,39,43,45]; Perforation [42,43]; Abscess [43,44]	Mucosal ulceration [31,33,34,36,38,43,51,52]; Granulation tissue [31,45]; Necrosis [33,35,42,43]; Edema [33]; Hemorrhage [33,36,38,43]; Infiltrating organisms in an inflammatory depleted background [33,36,38,39,43,52]; Microabscess [34]; Thrombosis [36,52]; Pseudomembranes [43]

## 6. Conclusions

Despite the recent interest and the growing number of publications targeting sepsis, intending to identify biomarkers useful both for its diagnosis, prognosis, and for the understanding of its pathogenesis, and especially for the multi-organ dysfunction, and despite the extensive research period of the literature review, the number of publications on the topic “neutropenic enterocolitis and sepsis” appears to be very small. In any case, the extrapolated data allowed us to conclude that the integration of medical history, clinical and laboratory data, radiological imaging, and macroscopic and histological investigations allowed us to identify a specific pathological profile. As regards the medical history, particular attention must be paid to subjects with onco-hematological neoplasia (in particular leukemia or solid tumors), in treatment with chemotherapeutic agents (among which particular attention must be paid to therapeutic schemes involving taxanes) [13,14,45]. An important clinical element is the triad composed of fever, abdominal pain, and diarrhea, but certainly the alarm bell is represented by marked neutropenia. In the case described, in fact, the patient had a history of solid tumor in treatment with poli-chemotherapy, according to an administration regimen that included taxanes, and entered the local hospital for fever, abdominal pain, and diarrhea. The most significant finding was the very low neutrophil count, which, as per the literature review, is more frequently associated with a worse prognostic characterized by hemodynamic instability and sepsis, which subsequently occurred. In the present case as in similar ones, a rapid diagnostic classification is essential to implement the earliest support measures, although based on the available data, neutropenic enterocolitis is characterized by high mortality even at early stages.

In this context, the case under examination is perfectly in line with the data from the literature review and seems to be an overlap of what Cornely and Schirmacher have already reported [52]. Even in our case, the demonstration of an altered intestinal mucosal barrier would seem to support the hypothesis of translocation as a prerequisite for subsequent bacteremia, sepsis, and multi-organ failure. Further studies are needed to better understand these aspects and if there are risk factors more correlated to a bad outcome.

## Data Availability

Data available on request due to restrictions e.g., privacy or ethical reason.
